# First person – Sevda Boyanova

**DOI:** 10.1242/dmm.052608

**Published:** 2025-08-26

## Abstract

First Person is a series of interviews with the first authors of a selection of papers published in Disease Models & Mechanisms, helping researchers promote themselves alongside their papers. Sevda Boyanova is first author on ‘ Multi-modal comparative phenotyping of knock-in mouse models of frontotemporal dementia/amyotrophic lateral sclerosis’, published in DMM. Sevda is a postdoc in the lab of Dr Frances Wiseman at University College London, London, UK, investigating behavioural analysis of mouse models of aspects of dementia, including frontotemporal dementia and Alzheimer's disease, to better understand the mechanisms of these diseases.


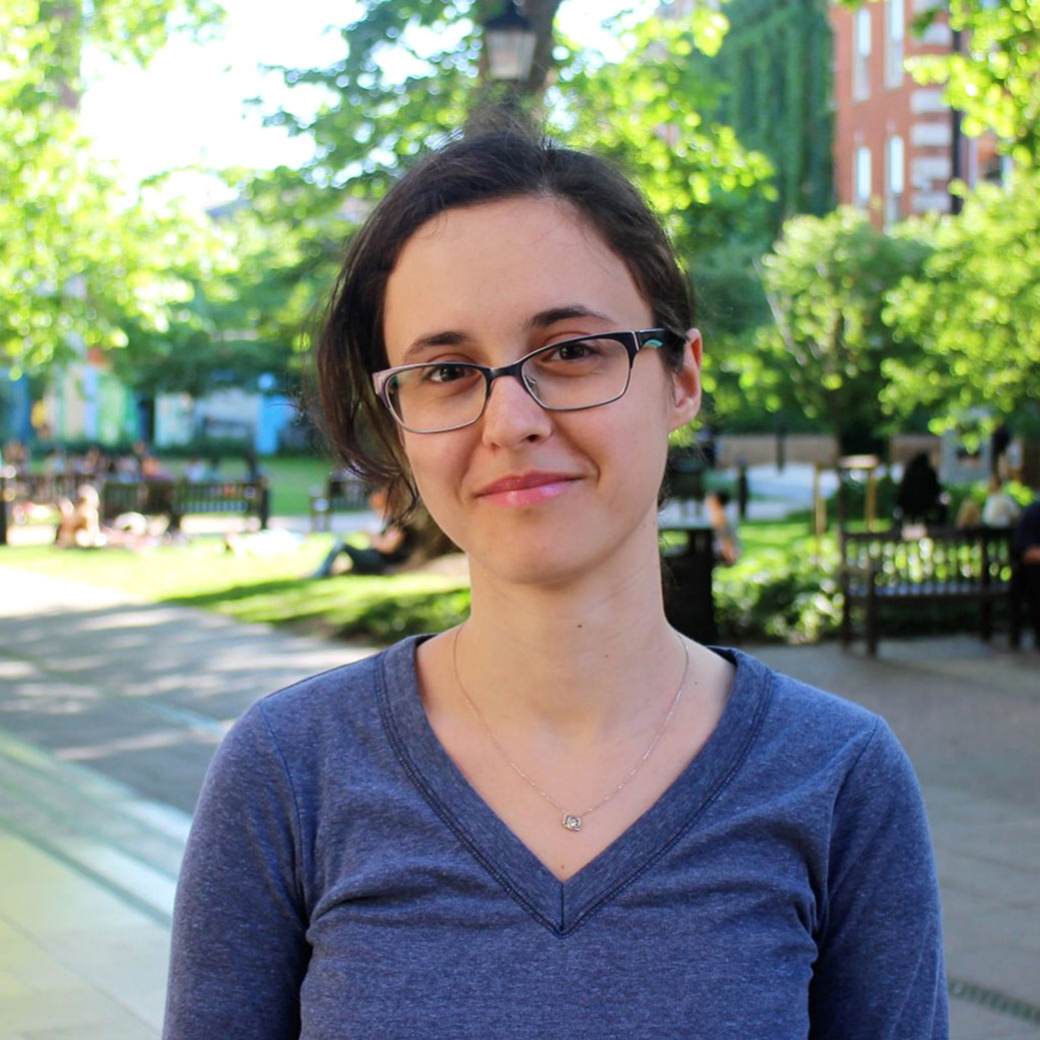


**Sevda Boyanova**

**Who or what inspired you to become a scientist?**

I have always been curious about wildlife and the living world, so biology was my favourite subject at school. However, in my teenage years, I became really fascinated by psychology and the brain. I wanted to understand why people behave in one way or another, what makes someone a genius musician or a brilliant mathematician, and why mental and neurological disorders arise. Reading some of the books by Oliver Sacks further strengthened these interests. Then, I found out about neuroscience, and I realised that would be the perfect subject to pursue at university – one that could help me start answering some aspects of these complex questions.

**What is the main question or challenge in disease biology you are addressing in this paper? How did you go about investigating your question or challenge?**

Currently, there are no complete animal models of amyotrophic lateral sclerosis/frontotemporal dementia (ALS/FTD). Therefore, assessing the newest next-generation knock-in models carrying mutations involved in these diseases in a systematic way and side by side is extremely important to understand their strengths and weaknesses. In our study, we ran a standardised longitudinal sensory, behavioural and cognitive evaluation of two knock-in mouse models of aspects of ALS/FTD (*C9orf72^GR400/+^* and *Tardbp^Q331K/Q331^*). We used behavioural assays to test the memory, social preference, anxiety-like behaviour and sensory modalities of the mice. This allowed us to see the effects of each mutation on its own and also to compare the two models in a meaningful way. For the *C9orf72^GR400/+^* model, we also investigated the effect of parental genotype on the behaviour of the offspring, which is not often included in the behavioural analysis of mouse models, and here I show it is an important factor to consider.

**How would you explain the main findings of your paper to non-scientific family and friends?**

Developing treatments for dementia is extremely challenging because there are many types of dementia and each one is very complex. Some types are caused by mutations that are inherited within a family. However, we do not always understand exactly how these mutations lead to disease. This is where mice can help us. We can insert the same mutation as the one that causes disease in people into the mice. Then we can test the mouse's behaviour. In the case of dementia, we might want to test memory. For example, we can check if mice with the mutation will remember a place less well than mice without the mutation. In our study, we compared the effect of two of these mutations side by side. We saw that the mice with different mutations had different changes in behaviour. Thus, these mice are useful to understand different aspects of frontotemporal dementia. This is very important because understanding the effects of different mutations in mice can help us understand disease mechanisms better and test future medications that could help treat patients.

[…] understanding the effects of different mutations in mice can help us understand disease mechanisms better and test future medications that could help treat patients

**What are the potential implications of these results for disease biology and the possible impact on patients?**

Our study provides new insight into genotype-phenotype relationships in the *C9orf72^GR400/+^* and *Tardbp^Q331K/Q331^* models. In the *C9orf72^GR400/+^* mice, we observed age-related deficit in short-term memory and that parental genotype affects exploration activity in offspring. In the *Tardbp^Q331K/Q331K^* model, we found age-related changes in weight, fat mass, locomotion and how the mice buried marbles. We did not find deficits in vision or olfactory habituation-dishabituation in either model. These results are important because they can inform choice of models for future studies into the disease mechanism or development of treatments and biomarkers.

**Figure:**
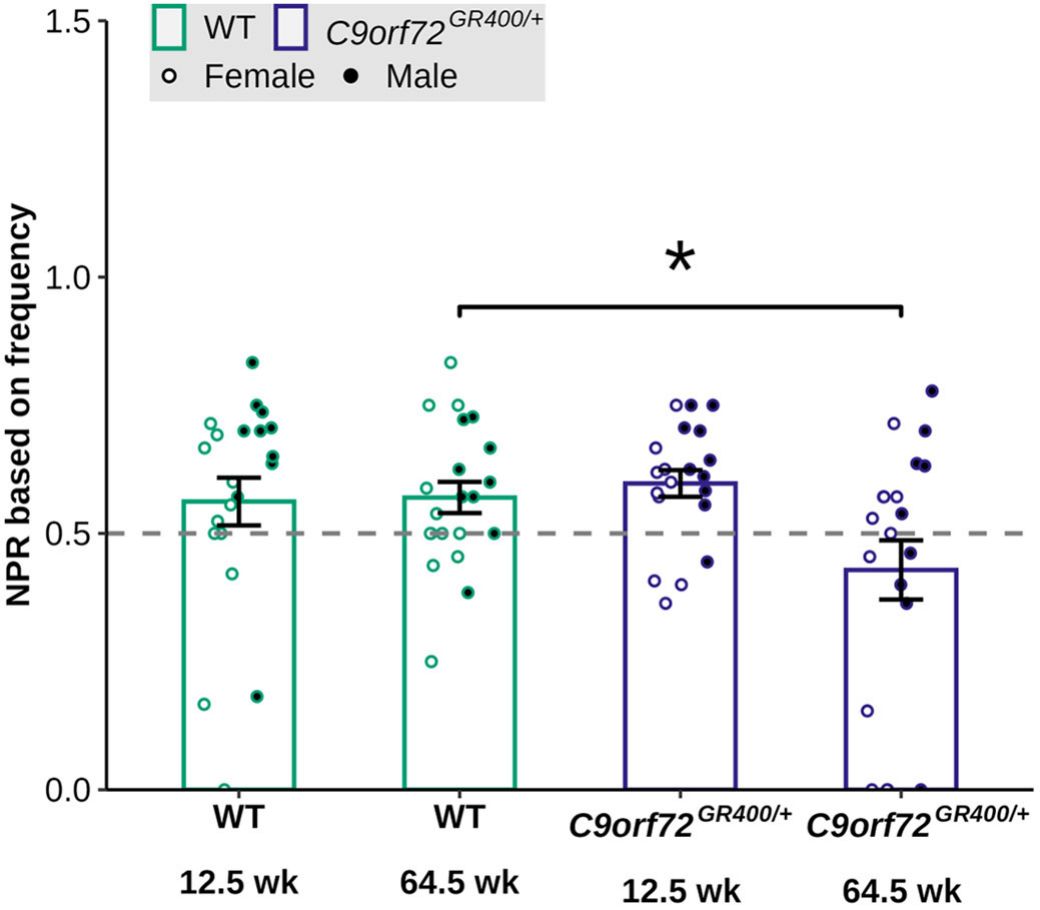
***C9orf72^GR400/+^* mice (purple) perform less well than controls (green) at 64.5 weeks of age in a short-term spatial memory test using a Y-shaped maze.** NPR, novel preference ratio.

**Why did you choose DMM for your paper?**

We believe our work showcasing a systematic study and comparison of two mouse models carrying mutations involved in ALS/FTD is well suited within the scope of DMM and will be of interest to its readers. Also, we appreciate that DMM is a well-respected and open source journal, as well as DMM's commitment to supporting early career researchers through their grants and fellowship programmes.

**Given your current role, what challenges do you face and what changes could improve the professional lives of other scientists in this role?**

I think most early-career researchers are affected by the instability of research funding. Having a bit longer time to follow through an idea or improve a certain skillset would be very helpful when transitioning to more independent research.

**What's next for you?**

I would love to advance my understanding and application of machine learning models to video analysis of mouse behaviour, especially in the home cage setting. I believe this is a great tool for deep understanding of mouse models, which also goes hand in hand with the highest standards of animal welfare.

**Tell us something interesting about yourself that wouldn't be on your CV**

I love travelling, finding local recipes that are new for me and then trying to cook them at home. You can learn a lot about different cultures and countries through their food.
